# Real‐time analysis of conformational control in electron transfer reactions of human cytochrome P450 reductase with cytochrome *c*

**DOI:** 10.1111/febs.13501

**Published:** 2015-09-16

**Authors:** Tobias M. Hedison, Sam Hay, Nigel S. Scrutton

**Affiliations:** ^1^ Manchester Institute of Biotechnology and Faculty of Life SciencesUniversity of ManchesterUK

**Keywords:** cytochrome P450 reductase, diflavin oxidoreductase, electron transfer, Förster resonance energy transfer, protein domain dynamics

## Abstract

Protein domain dynamics and electron transfer chemistry are often associated, but real‐time analysis of domain motion in enzyme‐catalysed reactions and the elucidation of mechanistic schemes that relate these motions to the reaction chemistry are major challenges for biological catalysis research. Previously we suggested that reduction of human cytochrome P450 reductase with the reducing coenzyme NADPH is accompanied by major structural re‐orientation of the FMN‐ and FAD‐binding domains through an inferred dynamic cycle of ‘open’ and ‘closed’ conformations of the enzyme (*PLoS Biol*, 2011, e1001222). However, these studies were restricted to stopped‐flow/FRET analysis of the reductive half‐reaction, and were compromised by fluorescence quenching of the acceptor by the flavin cofactors. Here we have improved the design of the FRET system, by using dye pairs with near‐IR fluorescence, and extended studies on human cytochrome P450 reductase to the oxidative half‐reaction using a double‐mixing stopped‐flow assay, thereby analysing in real‐time conformational dynamics throughout the complete catalytic cycle. We correlate redox changes accompanying the reaction chemistry with protein dynamic changes observed by FRET, and show that redox chemistry drives a major re‐orientation of the protein domains in both the reductive and oxidative half‐reactions. Our studies using the tractable (soluble) surrogate electron acceptor cytochrome *c* provide a framework for analysing mechanisms of electron transfer in the endoplasmic reticulum between cytochrome P450 reductase and cognate P450 enzymes. More generally, our work emphasizes the importance of protein dynamics in intra‐ and inter‐protein electron transfer, and establishes methodology for real‐time analysis of structural changes throughout the catalytic cycle of complex redox proteins.

**Abbreviations**
CPR: cytochrome P450 reductase
CYP: cytochrome P450
cyt *c*: cytochrome *c*
ET: electron transfer
FRET: Förster resonance energy transfer

## Introduction

Protein dynamics may be described in a similar way to protein folding, using a multi‐dimensional conformational landscape 1. Conformational landscapes encompass hill and valley features: the valley topographies represent the low‐energy states, while the hill landscapes correspond to the thermodynamic barriers between them. Use of spectroscopic and crystallographic measurements has shown that energy landscapes may be altered by pressure, temperature, mutagenesis and substrate/inhibitor binding, demonstrating that the study of conformational change is essential to understanding of the functionality of some proteins 2, 3, 4. However, it is difficult to study the conformational landscape of a protein as these landscapes encompasses a broad range of time scales (10^−12^ to > 1 s) and distance scales (10^−2^ to > 10 Å). The role of large‐scale domain dynamics that ‘gate’ biological electron transfer (ET) has been demonstrated in a number of enzyme families 5, 6. Such domain dynamics have been inferred, or directly shown, to be coupled to ET in a number of related diflavin oxidoreductases, including nitric oxide synthase 7, 8, 9, methionine synthase reductase 10, cytochrome P450 BM3 11, sulfite reductase 12 and cytochrome P450 reductase (CPR) 13, 14. Real‐time analysis of conformational change during catalysis in such complex redox proteins is a major challenge due to the rich optical properties of multi‐centre redox proteins and the inherent complexity of the enzyme catalytic cycle.

**Scheme 1:**
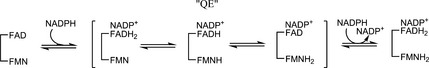
The reductive half‐reaction of CPR. NADPH binds to oxidized CPR, and a hydride anion is transferred from the reducing nicotinamide coenzyme NADPH to FAD. After FAD hydroquinone formation, electrons are ‘equilibrated’ across the FAD and FMN cofactors to form a quasi‐equilibrium state (‘QE’). With excess NADPH, further reduction occurs, leading to formation of an enzyme species that contains FAD and FMN hydroquinone forms.

CPR is a microsomal membrane‐anchored oxidoreductase that transfers electrons from NADPH to a variety of haem‐containing partner proteins, including cytochrome P450s (CYPs) 15, haem oxygenase 16, squalene monooxygenase 17 and cytochrome *c* (cyt *c*) 18. CPR contains two distinct redox domains, one housing an FAD cofactor and the other housing an FMN cofactor, which are separated by a flexible hinge region 19, 20, 21. The chemical mechanism of the reaction catalysed by CPR is complex (Scheme 1), but is well documented and involves binding of NADPH to the FAD domain, followed by hydride transfer from the nicotinamide to the N5 of the FAD 22. Following FAD hydroquinone formation, the FMN is reduced by intramolecular ET, and once one‐ or two‐electron‐reduced, may act as a one‐electron donor for microsomally bound haem proteins such as CYPs 23. The oxidized FAD may be further reduced by a second NADPH 22, and five oxidation states of CPR may be catalytically relevant: the fully oxidized and one‐, two‐, three‐ and four‐electron‐reduced forms 24. Cytochrome *c* may also act as a surrogate electron acceptor, and, like CYPs, accepts electrons from the reduced FMN cofactor 18. Crystallographic data show that the two flavin cofactors of CPR are positioned with a 4 Å edge‐to‐edge distance between the isoalloxazines 19, 21. Based on ‘Dutton's ruler’, this short distance should allow very rapid inter‐flavin ET (approximately 10^−10^ s^−1^) 25, 26, 27. However, temperature‐jump perturbation spectroscopy 28 and laser‐flash photolysis experiments 29, 30 have shown that ET between FAD and FMN co‐factors is relatively slow (approximately 10 s^−1^), suggesting that the inter‐flavin ET is adiabatic or ‘gated’. Furthermore, pH‐dependent and kinetic isotope studies have shown that the reductive half‐reaction catalysed by CPR is not gated by chemical steps (proton transfer) 24, which suggests that conformational changes/protein dynamics are likely to control the rate of inter‐flavin ET.

Multiple spectroscopic techniques, including mass spectrometry 31, NMR 32, small‐angle X‐ray scattering 32, neutron scattering 33 and reflection anisotropy 34, as well as construction of an ‘open’ yeast/human chimeric protein 35, have been used to demonstrate that CPR exists as a mixture of ‘open’ and ‘closed’ conformations, with long and short flavin–flavin distances, respectively. Moreover, pulsed electron–electron double resonance (PELDOR) spectroscopy measurements of two‐electron‐reduced (di‐semiquinone) forms of several diflavin oxidoreductases has shown that their conformational landscapes are ‘rugged’, with multiple conformers present 7, 10, 36. Many of these aforementioned studies may be interpreted as indicating that perturbation through coenzyme addition, redox state alterations and changes in ionic strength lead to shifts in the conformational landscapes of the enzyme. Further, both the deletion of a hinge region (ΔTGEE) 37 and chemical cross‐linking of thiols 38 in variant forms of CPR have shown that inter‐flavin ET is inhibited in the ‘open’ form of the enzyme, while a ‘closed’ cross‐linked form is unable to efficiently reduce cyt *c*. These data, coupled with studies of ET as a function of solvent viscosity, temperature and pressure 36, have highlighted the potential functional importance of redox‐driven conformational exchange between closed and open sub‐states of CPR for inter‐flavin ET and subsequent reduction of CYPs. However, these studies do not provide a direct ‘read‐out’ of real‐time conformational change concurrent with catalysis.

We recently reported an analysis of conformational change in CPR in the reductive half‐reaction (reduction of the flavins by NADPH) using stopped‐flow Förster resonance energy transfer (FRET) studies 14. The study was not extended to the complete reaction cycle, as this was not possible using a single‐mixing stopped‐flow method. Our previous study indicated coupling of dynamics to chemical catalysis during flavin reduction. However, the quenching of fluorescence emission prevented detailed analysis of the conformational transitions, such as direct spatial mapping of transitions from open to closed forms, and vice versa.

Here we address the limitations of the previous work. Specifically, we have developed a double‐mixing stopped‐flow method that enables real‐time analysis of conformational change throughout the complete catalytic cycle (reductive and oxidative half‐reactions). The limitations imposed by fluorescence quenching are largely overcome by use of longer‐wavelength FRET pairs. Use of these new FRET pairs enabled analysis of the oxidative half‐reaction (and thus also the complete reaction cycle) with the soluble electron acceptor cyt *c*, which is widely used as an electron acceptor in turnover studies of diflavin oxidoreductases 23, 39, including CPR 22, 40, 41. This methodology allowed the UV‐Vis kinetics of CPR redox chemistry to be followed concurrently with a time‐based analysis (approximately 5 ms to 10 s) and spatial analysis (20–80 Å) of CPR domain dynamics.

## Results

The rationale behind the present study was to establish a simple FRET model that reports directly on protein domain dynamics (i.e. opening and closing of CPR) and is not compromised by fluorescence changes attributed to quenching by the redox cofactors. Having established a suitable model, we set out to develop a double‐mixing stopped‐flow assay that can access the kinetics of ET from CPR to the soluble redox acceptor cyt *c*, which, together with previous studies of the reductive half‐reaction 14, provides a complete analysis of ET for the whole catalytic cycle. The new FRET model and double‐mixing stopped‐flow assays were then combined to investigate the real‐time dynamic changes throughout the CPR/cyt *c* catalytic cycle.

### FRET model of CPR domain dynamics

**Figure 1:**
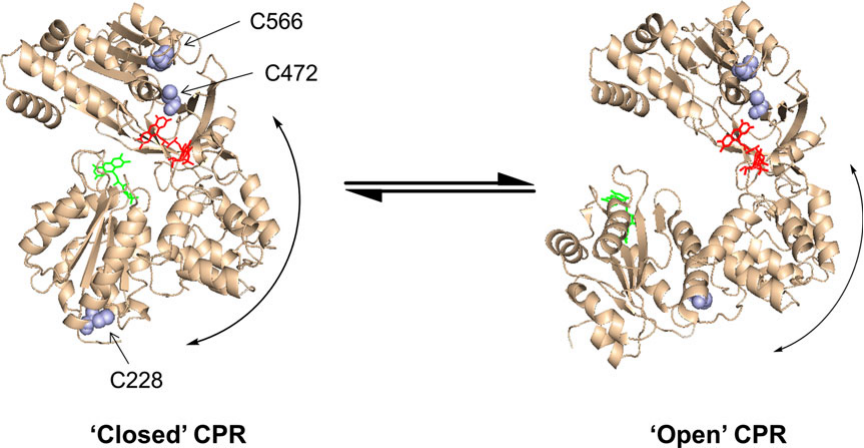
‘Open’ and ‘closed’ conformations of rat CPR. The open structure was generated from PDB ID 3ES9_A and the closed structure was generated from PDB ID PDB 1AMO_A. The C228–C556 and C228–C472 sulfur–sulfur distances are 46.9 and 51.5 Å, respectively, in the open form, and 51.2 and 57.3 Å, respectively, in the closed form, i.e. differences of 4.3 and 5.8 Å for the C228–C556 and C228–C472 sulfur–sulfur distances, respectively. The distances between C472 and C556 on the FAD‐binding domain are 14.4 and 14.7 Å for 1AMO_A and 3ES9_A, respectively. Both of these distances are too short to be observed by traditional FRET measurements, and may be ignored in this investigation.

**Figure 2:**
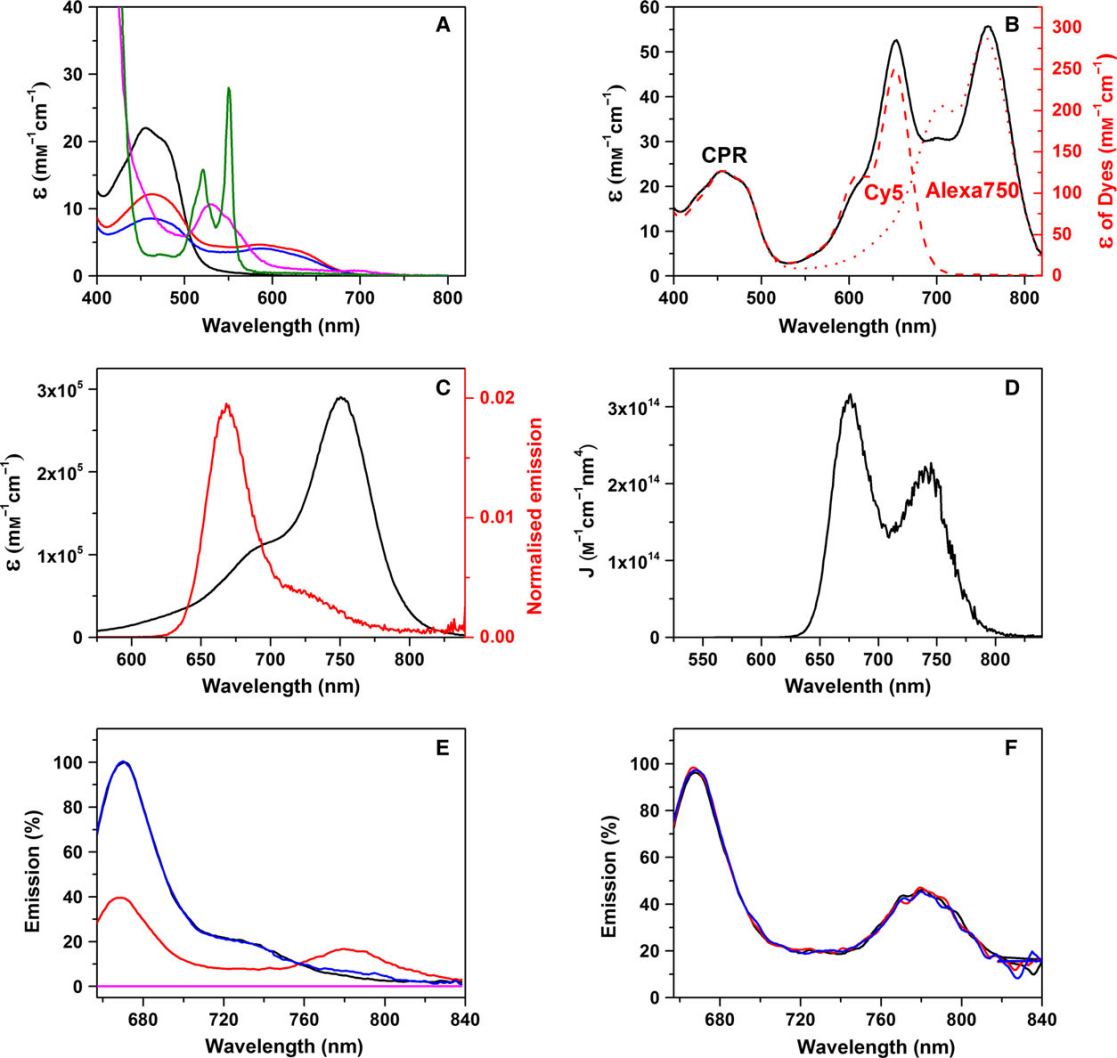
Absorption and fluorescence spectra of CPR‐DA. (A) Absorption spectra for oxidized CPR (black), CPR reduced with one equivalent of NADPH (red) or 20 equivalents of NADPH (blue), oxidized cyt *c* (magenta) or sodium dithionite‐reduced cyt *c* (green). (B) Absorption spectra for Cy5 and Alexa750 bound to CPR in an approximately 1 : 1 ratio (black), and for CPR singly labelled with Cy5 (red dashed line) or Alexa750 (red dotted line). (C) UV‐Vis spectrum for Alexa 750 (black) and emission spectrum for Cy5 excited at 560 nm (red). (D) Calculated spectral overlap for the spectra shown in (C). The spectral overlap gives a Förster radius, *R*_0_, of 68 Å, calculated as *R*_0_ = 0.2108[κ^2^ Φ_0_ *n* ^−4^ *J*]^1/6^, where κ^2^ = 2/3, Φ_0_ = 0.28, *n *=* *1.33 and *J *=* *2.40 × 10^13^ m ^−1^·cm^−1^·nm^4^. (E) Fluorescence emission spectra for CPR‐D (black), CPR‐DA (red), an equal mix of CPR‐D and CPR‐A (blue), and unlabelled CPR (magenta) when excited at 650 nm. These spectra are normalized to 670 nm, and the emission of the doubly labelled sample is scaled for CPR concentration. (F) Fluorescence emission spectra for CPR‐DA (black) and CPR‐DA mixed with a 20‐fold excess of reduced cyt *c* (red) or oxidized cyt *c* (blue) (20 μm), when excited at 650 nm. Experimental conditions were typically 1 μm labelled CPR in 50 mm potassium phosphate buffer (pH 7.0) at 25 °C.

We took advantage of naturally occurring cysteine residues in human CPR (C228, C472 and C566) to attach the fluorophores for FRET analysis. Using mass spectrometry, we showed previously that these residues are labelled readily 14. Although there are two accessible cysteine residues in the FAD‐binding domain (C472 and C566), modification of one prevents labelling at the other due to their proximity. Both residues are similarly separated from C228, and, as discussed previously, this simplifies the FRET analysis 14. The location of these cysteine residues dictates that the distance between fluorophore dyes is changes as CPR adopts more open or closed conformations (Fig. 1). In Fig. 2A, the absorbance spectra of CPR and cyt *c* in various states of reduction are shown. As the spectral features of the heam and flavin cofactors bound to CPR and cyt *c* span the UV and most of the visible spectrum, these two chromophores may cause significant quenching of dye fluorescence emission. Therefore, the fluorescence labels Cy5 (donor, D) and Alexa 750 (acceptor, A), which emit in the far‐red/near‐IR region, and are thus unlikely to be significantly quenched by haem or flavin, were selected to monitor domain dynamics in this investigation (Fig. 2B).

Binding of the Cy5/Alexa 750 pair to CPR (two dyes per protein, referred to as CPR‐DA) was shown to have an efficiency of 19 ± 3% (eight experiments), based on the extinction coefficients of dyes and protein (Fig. 2B). Moreover, from the absorption and emission spectra, the pair was calculated as having a Förster radius (*R*_0_) of approximately 68 Å (Fig. 2C,D), which is slightly larger than the inter‐cysteine (S–S) distances calculated from the ‘open’ and ‘closed’ conformers of rat CPR (94% sequence homology to human CPR), which are 51.2 and 57.3 Å in the closed form and 46.9 and 51.5 Å in the open form (Fig. 1) 19, 37. However, the distances between the dyes in the various conformers of CPR are likely to be greater than inter‐cysteine distances due to the size of the linker between the cysteine and the fluorescent moiety present on the maleimide labels (approximately 10 Å). Thus, as the dye‐dye distance is likely to be similar to *R*_0_, the Cy5/Alexa 750 dye pair is expected to be sensitive to conformational changes associated with domain dynamics in CPR.

Figure 2E shows the fluorescence emission spectra of CPR‐D (donor‐labelled) and CPR‐DA when excited at 650 nm (donor excitation maximum). A significant change in emission at 670 and 780 nm was observed for CPR‐DA, corresponding to a decrease in donor emission and an increase in acceptor emission compared with the CPR‐D spectrum. These changes in fluorescence emission are indicative of FRET from donor to acceptor fluorophores. Furthermore, when an equimolar mixture of singly labelled CPR‐D and CPR‐A (acceptor‐labelled) is excited at 650 nm, negligible FRET is observed. This lack of significant inter‐molecular FRET from CPR‐D to CPR‐A suggests that, as expected, there is no significant dimerization of oxidized CPR. Also, when non‐labelled CPR was excited at 650 nm, minimal intrinsic flavin fluorescence was observed. Figure 2F shows the emission spectra of CPR‐DA in the presence of 20 μm oxidized cyt *c* or 20 μm reduced cyt *c*. The lack of change in dye emission indicates there is no quenching of dye fluorescence by the haem present in cyt *c*. Combined, the data indicate that labelling of CPR using the long‐wavelength dye pair Cy5/Alexa 750 gives rise to a FRET signal that is probably responsive to distance changes, and is not affected by cofactor quenching of the signal or inter‐molecular FRET.

### Double‐mixing stopped‐flow assays of ET from NADPH‐reduced CPR to cyt *c*

#### Double‐mixing assay for cyt *c* reduction

Before monitoring redox‐linked domain dynamics of CPR, the oxidative kinetics of the enzyme were studied by pre‐steady state UV‐Vis stopped‐flow spectroscopy. A double‐mixing regime was developed to follow the reduction of cyt *c* by NADPH‐reduced CPR. The double‐mixing stopped‐flow method allowed rapid mixing of CPR and NADPH in the first mixing event. This leads to reduction of CPR; with one stoichiometric equivalent of NADPH, the end point is a mixture of reduced CPR forms determined by the potentials of the flavin and nicotinamide coenzyme couples (Scheme 1). After the first mix, the reduced forms of CPR were then incubated in the ageing loop of the stopped‐flow spectrometer for various ageing times before finally being mixed with oxidized cyt *c*.

**Figure 3:**
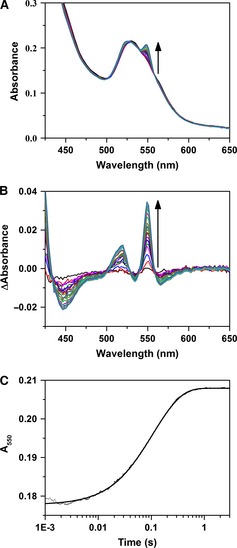
Cytochrome *c* reduction by CPR. (A) Stopped‐flow photodiode array spectra showing cyt *c* reduction by 1× NADPH‐reduced CPR. (B) Absorbance difference spectra, relative to the spectrum at 5 ms, derived from the data in (A). (C) Single wavelength transient at 550 nm derived from the data in (A) fitted to a double‐exponential function. Experimental conditions were 2 μm CPR mixed with 2 μm NADPH and aged for 2 s prior to a second mix with 20 μm cyt *c*. All samples were in suspended in 50 mm potassium phosphate buffer (pH 7.0) at 25 °C.

Photodiode array spectra were initially recorded to follow the reaction of cyt *c* with NADPH‐reduced CPR. The photodiode array data, shown in Fig. 3A, indicate that the oxidation of CPR may be followed by monitoring the rapid increase in absorbance of the haem β‐band at 550 nm. Transients monitoring the reduction of cyt *c* at 550 nm were found to fit to a double exponential function (Fig. 3C). Furthermore, under the stated conditions, the 600 nm feature (Fig. 3A) of the flavin neutral di‐semiquinone has a minimal spectral contribution (Fig. 3B).

#### Optimizing the ageing time between stoichiometric reduction of CPR and subsequent reduction of cyt *c*

**Figure 4:**
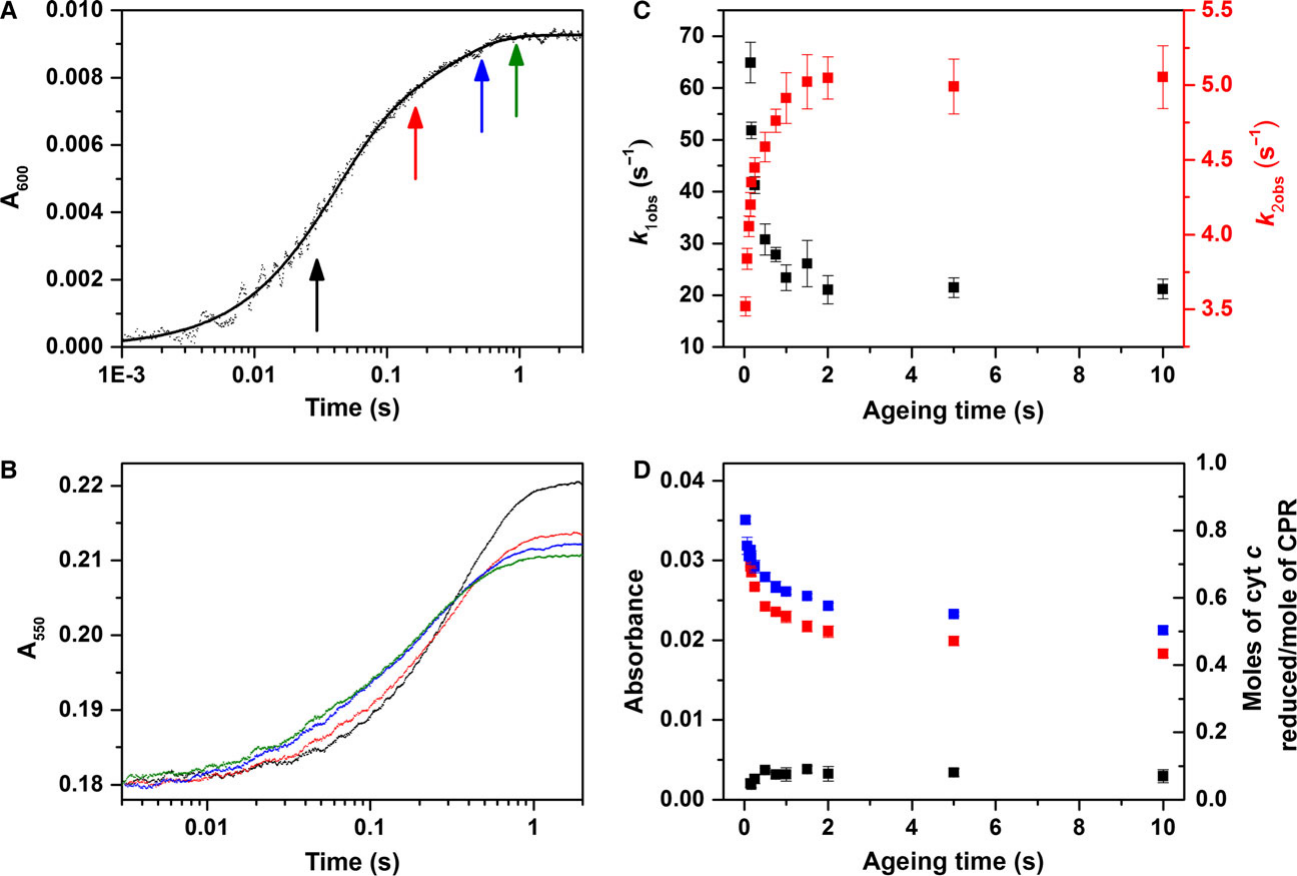
Cytochrome *c* reduction by CPR. (A) Stopped‐flow transient monitoring flavin reduction in CPR by equimolar NADPH (4 μm). (B) Double‐mixing stopped‐flow transients showing the dependence of cyt *c* reduction by 1× NADPH‐reduced CPR on ageing time. The colour of each trace represents the ageing time as indicated by coloured arrows in (A). Transients monitoring cyt *c* reduction by 1× NADPH‐reduced CPR were measured at 600 nm, and fitted to a double‐exponential function. (C, D) The dependence of the observed rate constants and amplitudes (*A*_*i*_, Eqn (2)) on ageing time is shown in (C) and (D), respectively. For the amplitude data, the first kinetic phase is shown in black, the second in red, and the sum in blue. Experimental conditions are equivalent to those in Fig. 3, with ageing times between 0.03 and 10 s.

Optimal double‐mixing conditions were established by varying the ageing time after mixing of equimolar amounts of oxidized CPR and NADPH. Figure 4A shows an example transient of the first mix in the stopped‐flow experiment: stoichiometric reduction of CPR by NADPH (denoted 1 × NADPH‐reduced CPR), followed by monitoring the neutral semiquinone species at 600 nm. The trace was shown to fit to a double‐exponential function over the 4 s time scale recorded, with rate constants for the first and second phases of 30.0 ± 1.7 (64%) and 4.6 ± 0.5 s^−1^ (36%), respectively. While these apparent rate constants are likely to be a convolution of multiple steps, the faster step probably reflects the initial ET from FAD hydroquinone to FMN, with the slower phase reflecting establishment of the ‘quasi‐equilibrium’ species shown in Scheme 1. After the first mix, the two‐electron‐reduced CPR present in the ageing loop was subsequently mixed with ferric cyt *c* at various ‘ageing’ times, and cyt *c* reduction was monitored at 550 nm (Fig. 4B). These data were also fitted to a double‐exponential function, and Fig. 4C,D show how the observed rate constants and amplitude changes exhibit a hyperbolic dependence on the ageing time (∼1 s), saturating on the same timescale as the QE state is established. At earlier ageing times, before the two‐electron‐reduced CPR has equilibrated, the cyt *c* reduction kinetics are both faster and have higher yield, but the reaction kinetics are very sensitive to ageing time (the mechanistic reasons for which remain uncertain). As the observed rate constants and amplitude changes associated with the kinetic phases of CPR oxidation are invariant at ageing times > 1 s (Fig. 4C,D), and the largest population of the CPR di‐semiquinone species is formed during this time (Fig. 4A), a 2 s ageing time was selected for subsequent experiments.

As the concentration of reduced cyt *c* is readily determined from the fitted change in absorbance (amplitude) at 550 nm, the stoichiometry of the reaction was determined. At short ageing times, approximately one molecule of cyt *c* is reduced per CPR, with this efficiency decreasing at longer ageing times. This reaction stoichiometry suggests that the one‐electron‐reduced CPR does not reduce cyt *c* at this experimental time scale (4 s).

#### Double‐mixing studies with a fixed ageing time and variable NADPH concentration

**Figure 5:**
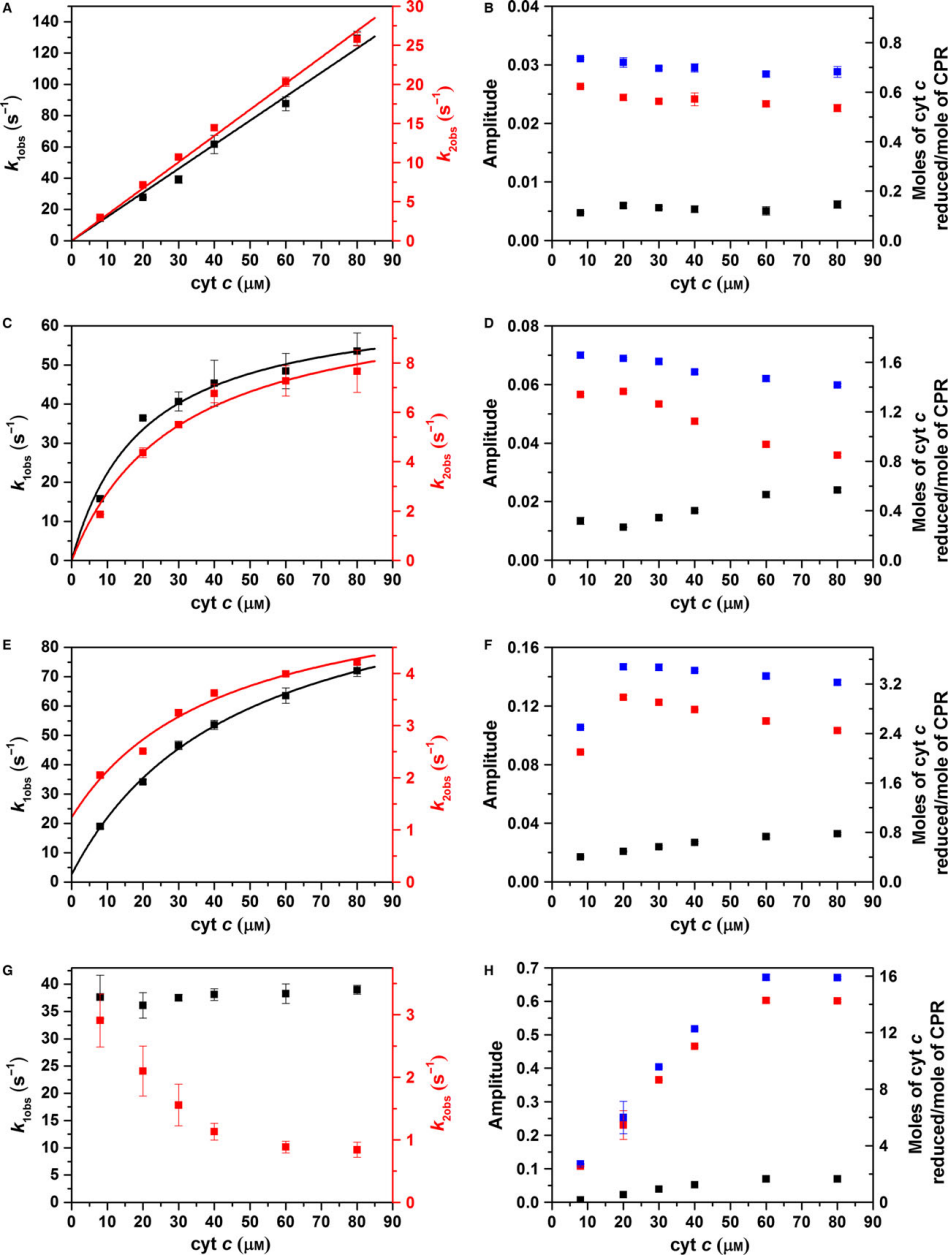
Cytochrome *c* reduction by CPR. The cyt *c* concentration dependence of the rate constants (A, C, E, G) and amplitudes (B, D, F, H) describing the reduction of cyt *c* by CPR reduced using equimolar NADPH (A, B), or twofold (C, D), fourfold (E, F) and 20‐fold (G, H) equivalents of NADPH in a double‐mixing stopped‐flow experiment with an ageing time of 2 s. For the amplitude data, the first kinetic phase is shown in black, the second in red, and the sum in blue. Experimental conditions are equivalent to those in Fig. 3, and data fitting is described in Table 1.

Figure 5 illustrates the kinetics of cyt *c* reduction by NADPH‐reduced CPR that has been incubated in the ageing loop for 2 s. When equimolar NADPH and CPR are mixed together (Fig. 5A,B), two second‐order kinetic phases are observed, with rate constants of 1.54 ± 0.04 and 0.33 ± 0.01 μm ^−1^·s^−1^. Alternatively, for experiments with 2 : 1 and 4 : 1 ratios of NADPH to CPR, two kinetic phases are observed, with the observed rate constants both saturating at higher cyt *c* concentrations (Fig. 5C,E). When CPR is reduced using a 20‐fold excess of NADPH (Fig. 5G), the observed rate constants from two kinetic phases are both largely independent of cyt *c* concentration; while the second phase does show a small decrease in observed rate constant, this probably arises from the poor fitting of the data due to complications resulting from multiple turnover of CPR (data not shown).

**Table 1:** Oxidative half‐reaction kinetics. Fitting parameters for the data in Fig. 5. The stoichiometric data demonstrated second‐order kinetics and were fitted to a linear function, while other data were fitted to Eqn (3)

Kinetic phase	Parameters	NADPH equivalents		
		1	2	4
1st	*k*_for_ (s^−1^)	–	66.9 ± 4.4	109.8 ± 5.1
	*k*_rev_ (s^−1^)	–	0^a^	2.6 ± 2.7
	*K*_S_ (μm)	–	19.9 ± 3.9	46.8 ± 8.1
	*k*_for_/*K*_S_ (μm ^−1^·s^−1^)	–	3.4 ± 0.9	2.3 ± 0.5
	*k* (μm ^−1^·s^−1^)	1.54 ± 0.04	–	–
2nd	*k*_for_ (s^−1^)	–	10.9 ± 0.9	4.7 ± 0.6
	*k*_rev_ (s^−1^)	–	0^a^	1.2 ± 0.4
	*K*_S_ (μm)	–	29.9 ± 6.2	42.2 ± 22.8
	*k*_for_/*K*_S_ (μm ^−1^·s^−1^)	–	0.37 ± 0.11	0.11 ± 0.07
	*k* (μm ^−1^·s^−1^)	0.33 ± 0.01	–	–

a Not significantly larger than zero so value fixed to 0.

The reaction stoichiometry was again determined by taking the sum of the fitted amplitudes of each kinetic phase (Fig. 5B,D,F,H), and, in all cases, cyt *c* was reduced in sub‐stoichiometric amounts relative to the added NADPH. These data suggest that some oxidation state(s) of CPR are unable to reduce cyt *c*, and/or electron flux through CPR (from NADPH to FAD to FMN) is slow relative to the experimental time scale (approximately 10 s). The pseudo steady‐state flux of electrons through CPR to cyt *c* (*V*_app_) was determined by calculating the fractional amplitude (*A*_*i*_)‐weighted sum of the rate constant (*k*_*i*_) for each stopped‐flow experiment as shown:

(1) $$V_{\mathrm{app}}=\Sigma_{i\;\;=1}^{n}A_{i}k_{i}.$$

**Figure 6:**
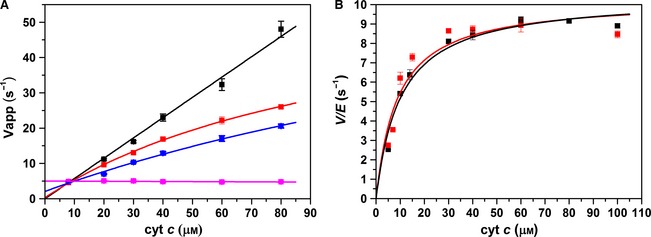
(Quasi) steady‐state kinetics. (A) Electron transfer flux during cyt *c* haem reduction by CPR reduced with equimolar NADPH (black) or twofold (red), fourfold (blue) and 20‐fold (magenta) equivalents of NADPH, determined by fitting the data in Fig. 5 to Eqn (1). Data are fitted to a polynomial function (solid lines) to guide the eye. (B) Steady‐state kinetics of 2 nm wild‐type CPR (black) and donor‐acceptor labelled CPR (red) with 100 μm NADPH and excess cyt *c*, measured by absorption at 550 nm. Experiments were performed in 50 mm potassium phosphate buffer (pH 7.0) at 25 °C. The data in (B) are fitted to a Michaelis–Menten equation with *k*_cat_ = 10.4 ± 0.4 s^−1^ and *K*_m_ = 10.2 ± 1.7 μm (wild‐type) and 10.3 ± 0.8 s^−1^ and 8.8 ± 2.5 μm (CPR‐DA).

When the NADPH concentration is low, the electron flux is dependent on cyt *c* concentration, whereas, at higher levels of NADPH, multiple turnovers of CPR are possible, and the electron flux becomes cyt *c*‐independent and approaches steady‐state levels: the electron flux with 20 : 1 NADPH:CPR is 4.9 ± 0.1 s^−1^, whereas the *k*_cat_ is 10.4 ± 0.4 s^−1^ (Fig. 6). As the electron flux is not slow during these stopped‐flow experiments, it appears that the sub‐stoichiometric reduction of cyt *c* probably arises from accumulation of one or more oxidation states of CPR over the experimental time scale that are unable to reduce cyt *c*.

### Measurements of CPR domain dynamics by stopped‐flow fluorescence spectroscopy

**Figure 7:**
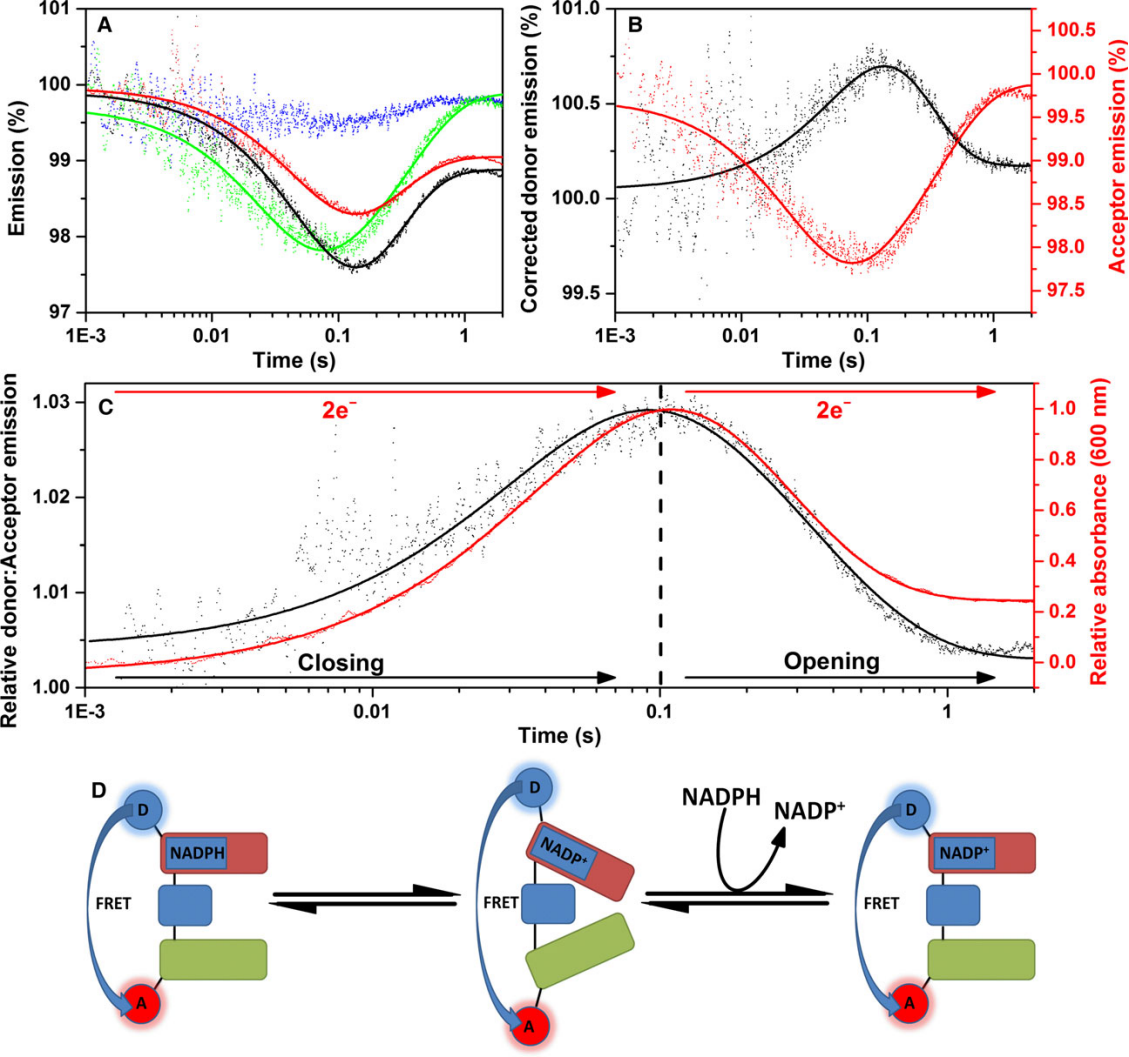
Stopped‐flow fluorescence of CPR‐DA versus 40‐fold excess NADPH. (A) Labelled CPR stopped‐flow fluorescence transients. Fluorescence donor transients are indicated in black for singly labelled protein (CPR‐D) and red for doubly labelled protein (CPR‐DA), whereas acceptor transients are indicated in blue for singly labelled protein (CPR‐A) and green for doubly labelled protein (CPR‐DA). (B) Acceptor and corrected donor emission. (C) CPR donor:acceptor ratio (representing domain dynamics) plotted against an example UV‐Vis transient, measured at 600 nm (CPR reaction kinetics). (D) Schematic of domain dynamics, showing the predominant conformational sub‐state during the reductive half‐reaction. The FAD/NADP ^+^‐binding domain, the FMN‐binding domain and the connecting domain are represented by red, green and blue rounded rectangles, respectively. Experimental conditions were 50 mm potassium phosphate buffer (pH 7.0), 1 μm labelled/15 μm unlabelled CPR, at 25 °C. Donor and acceptor fluorophores were excited at 650 and 750 nm, respectively, and fluorescence data were smoothed using a five‐point moving average.

The conformational landscape associated with the reductive half‐reaction of CPR has previously been studied using fluorescence stopped‐flow spectroscopy with fluorophore‐labelled enzyme (Alexa 488/Cy5) 14. These experiments were performed using excess NADPH, and demonstrated that domain dynamics are kinetically linked to reaction chemistry. Initially, a similar experiment was performed here using CPR labelled with Cy5 and Alexa 750 (CPR‐DA). The CPR‐DA exhibits similar UV‐Vis reaction kinetics to the unlabelled enzyme, with steady‐state *k*_cat_ and *K*_m_ values of 10.3 ± 0.8 s^−1^ and 8.8 ± 2.5 μm (the values for unlabelled enzyme are 10.4 ± 0.4 s^−1^ and 10.2 ± 1.7 μm; Fig. 6B), and observed reductive half‐reaction rate constants of 18.7 ± 0.7 and 4.3 ± 0.2 s^−1^ for the first and second NADPH reduction of CPR, respectively (the values for unlabelled protein are = 14.9 ± 0.1 and 4.0 ± 0.1 s^−1^). Figure 7A shows stopped‐flow transients monitoring the fluorescence emission (650 nm excitation of the donor) from the singly labelled enzymes CPR‐D and CPR‐A and the doubly labelled CPR‐DA enzyme upon reduction using a 40‐fold excess of NADPH. CPR‐D exhibits two distinct kinetic phases: the first shows fluorescence quenching, with a rate constant of 15.8 ± 0.7 s^−1^, while the second shows de‐quenching, with a rate constant of 4.3 ± 0.3 s^−1^. As there was no acceptor fluorophore present in this sample, it is likely that the quenching arises from conformational rearrangement of the enzyme and/or quenching by the flavin neutral (di)semiquinone species. The donor fluorescent transient for CPR‐DA displays a similar emission behaviour and kinetics to the CPR‐D (Table 2), but there is a reduction in the magnitude of dye quenching. This difference in fluorescence quenching between the doubly and singly labelled samples may possibly be attributed to FRET between the donor and acceptor fluorophore. CPR‐A (Fig. 7A) shows no changes in fluorescence emission when excited at 750 nm (a significant improvement over our previous study in which Cy5 was used as the acceptor 14), while the acceptor transient recorded for CPR‐DA upon 650 nm excitation exhibits two distinct kinetic phases that may also be attributed to FRET between the donor and acceptor fluorophore (Table 2). To interpret the FRET changes associated with protein dynamics only, the CPR‐DA donor emission was corrected by subtracting the CPR‐D emission 14. The deconvoluted FRET changes associated with the donor and acceptor fluorophores were fitted to a double‐exponential function (Fig. 7B). Moreover, the relative donor:acceptor (D:A) emission (FRET response) was calculated (Fig. 7C), and the data were fitted to a double‐exponential function with rate constants of 26.0 ± 0.8 and 3.1 ± 0.1 s^−1^. Based on crystal structures of ‘open’ and ‘closed’ forms of rat CPR 19, 37, we expect the D–A distance to reduce by approximately 5 Å when CPR opens (Fig. 1). The two kinetic phases in Fig. 7B,C may thus be attributed to closing and opening of CPR on the same time scale as the reaction kinetics, as illustrated in the schematic shown in Fig. 7D.

**Table 2:** Comparison of FRET and UV‐Vis stopped‐flow kinetics. Rate constants determined by fitting fluorescence transients in Figures 6, 7, 8 to Eqn (2). Flavin and cyt *c* reduction were monitored by absorbance at 600 and 550 nm, respectively, under the same conditions as fluorescence measurements

	*k*_1obs_ (s^−1^)	*k*_2obs_ (s^−1^)
CPR versus 40‐fold NADPH		
CPR‐D	15.8 ± 0.7	4.3 ± 0.3
CPR‐DA	18.0 ± 0.7	4.0 ± 0.3
Corrected CPR‐DA donor	11.8 ± 2.0	5.0 ± 1.0
CPR‐DA acceptor	36.0 ± 0.9	2.7 ± 0.1
D:A emission	26.0 ± 0.9	3.1 ± 0.1
Flavin reduction	14.9 ± 0.1	4.0 ± 0.1
CPR versus stoichiometric NADPH		
CPR‐D	34.1 ± 0.5	2.7 ± 0.1
CPR‐DA	27.7 ± 0.4	2.4 ± 0.1
Corrected CPR‐DA donor	ND	ND
CPR‐DA acceptor	47.5 ± 1.0	ND
D:A emission	47.9 ± 1.1	ND
Flavin reduction	30.6 ± 4.1	2.7 ± 0.2
1 : 1 NADPH:CPR versus 20 μm cyt *c*		
CPR‐D	133.3 ± 5.3	1.96 ± 0.02
CPR‐DA	110.9 ± 6.4	1.77 ± 0.02
Corrected CPR‐DA donor	1.2 ± 0.1	ND
CPR‐DA acceptor	ND	ND
D:A emission	0.53 ± 0.02	ND
cyt *c* reduction	22.7 ± 4.3	5.9 ± 0.2
1 : 1 NADPH:CPR versus 40 μm cyt *c*		
CPR‐D	146.5 ± 9.8	1.88 ± 0.03
CPR‐DA	178.2 ± 9.3	1.78 ± 0.02
Corrected CPR‐DA donor	1.6 ± 0.1	ND
CPR‐DA acceptor	ND	ND
D:A emission	0.43 ± 0.03	ND
cyt *c* reduction	43.1 ± 1.2	14.3 ± 0.3

ND, not detected.

**Figure 8:**
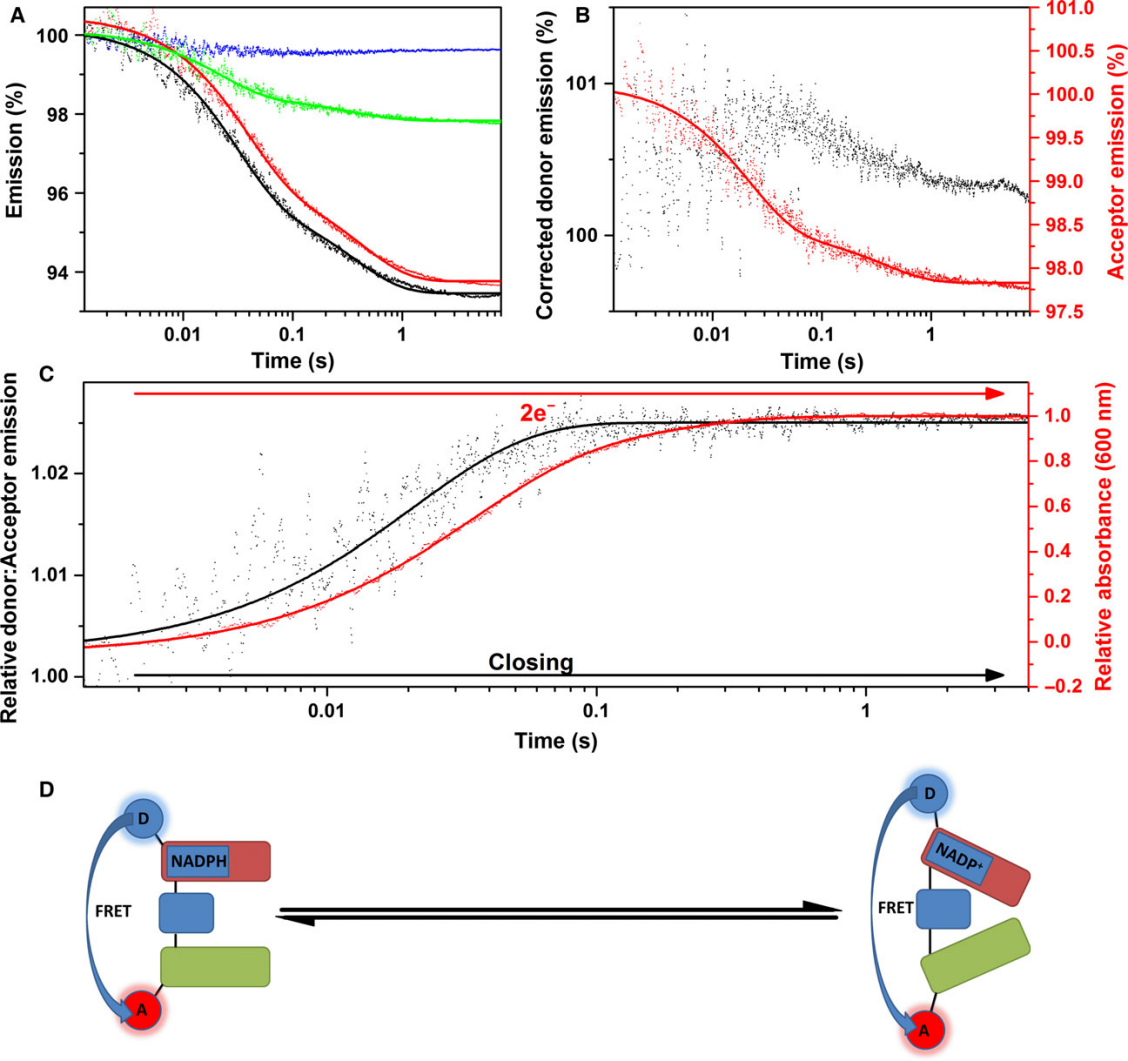
Stopped‐flow fluorescence of CPR‐DA versus stoichiometric NADPH. Labelled CPR stopped‐flow fluorescence transients. Fluorescence donor transients are indicated in black for singly labelled protein (CPR‐D) and red for doubly labelled protein (CPR‐DA), whereas acceptor transients are indicated in blue for singly labelled protein (CPR‐A) and green for doubly labelled protein (CPR‐DA). (B) Acceptor and corrected donor emission. (C) CPR donor:acceptor ratio (representing domain dynamics) plotted against an example UV‐Vis transient, measured at 600 nm (CPR reaction kinetics). (D) Schematic of domain dynamics showing the predominant conformational sub‐state during the reductive half‐reaction. The FAD/NADP ^+^‐binding domain, the FMN‐binding domain and the connecting domain are represented by red, green and blue rounded rectangles, respectively. Experimental conditions were 50 mm potassium phosphate buffer (pH 7.0), 1 μm labelled/15 μm unlabelled CPR, at 25 °C. Donor and acceptor fluorophores were excited at 650 and 750 nm, respectively, and fluorescence data were smoothed using a five‐point moving average.

Figure 8 shows the FRET changes associated with the reduction of CPR by stoichiometric NADPH. As also seen in studies using a 40‐fold excess of NADPH, the singly labelled CPR‐D shows a quenching of fluorescence over the time scale recorded (Fig. 8A). This same pattern of donor quenching is seen in the CPR‐DA sample, but the magnitude of quenching is reduced. The relatively small difference in emission between the CPR‐D and CPR‐DA samples may be attributed to FRET between the donor and acceptor dyes. The deconvoluted donor transient (Fig. 8B) cannot be adequately fitted by Eqn (2) due to the poor signal‐to‐noise ratio. However, as the CPR‐A sample (Fig. 8A) shows no change in fluorescence emission over the course of the experiment, emission from the CPR‐DA acceptor is likely to arise from FRET from the donor, and shows fluorescence quenching with two kinetic phases (Table 2). The D:A emission ratio shows a FRET change that may be attributed to closing of CPR, with a rate constant of 47.9 ± 1.1 s^−1^. These rate constants are similar to those obtained in UV‐Vis studies of the reductive half‐reaction of CPR when mixed with equimolar NADPH (Table 2).

**Figure 9:**
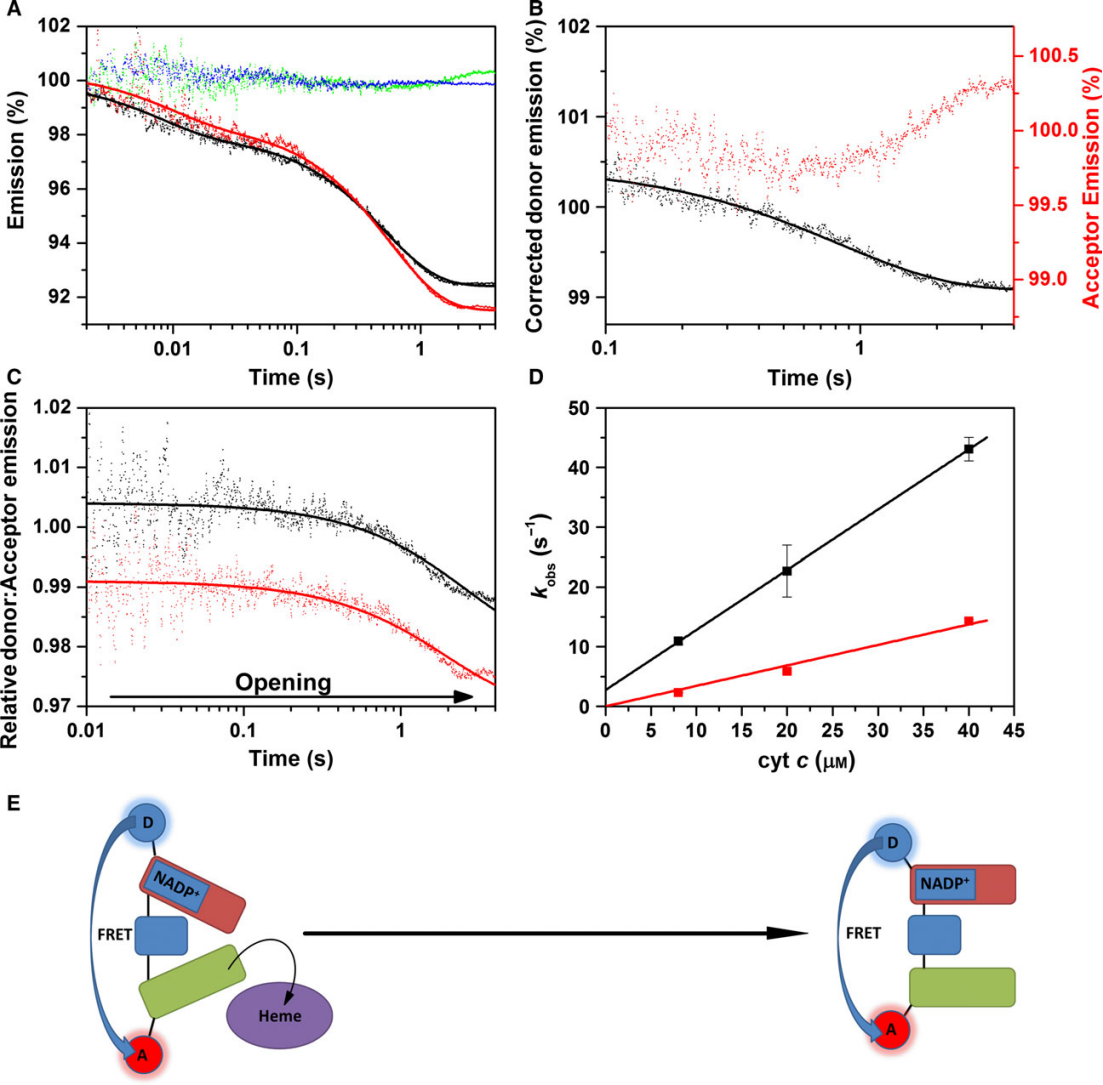
Double‐mixing stopped‐flow fluorescence of 1 × NADPH‐reduced CPR versus cyt *c*. (A) Labelled CPR stopped‐flow fluorescence transients. Fluorescence donor transients are indicated in black for singly labelled protein (CPR‐D) and red for doubly labelled protein (CPR‐DA), whereas acceptor transients are indicated in blue for singly labelled protein (CPR‐A) and green for doubly labelled protein (CPR‐DA). (B) Acceptor and corrected donor emission. (C) CPR donor:acceptor ratio (representing domain dynamics) for both reactions with 20 μm cyt *c* (black) and 40 μm cyt *c* (red). In (C), data are vertically offset to show the individual traces. (D) UV‐Vis kinetics of 1 × NADPH‐reduced CPR‐DA with cyt *c*. The black and red datasets represent the first and second kinetic phases, respectively. Both datasets are fitted to linear trends with second‐order rate constants of 1.01 ± 0.01 and 0.34 ± 0.02 μm ^−1^·s^−1^ for the first‐ and second‐order rate constants, respectively. (E) Schematic of domain dynamics showing the predominant conformational sub‐state during the oxidative half‐reaction of CPR. The FAD/NADP ^+^‐binding domain, the FMN‐binding domain and the connecting domain are represented by red, green and blue rounded rectangles, respectively. The redox partner protein cytochrome *c* is shown as a purple oval. Experimental conditions were 50 mm potassium phosphate buffer (pH 7.0), 1 μm labelled CPR (4 μm labelled CPR for UV‐Vis measurements), at 25 °C. Donor and acceptor fluorophores were excited at 650 and 750 nm, respectively, and fluorescence data were smoothed using a five‐point moving average.

Examples of fluorescence double‐mixing stopped‐flow transients monitoring the reduction of cyt *c* by equimolar NADPH‐reduced CPR are shown in Fig. 9A. The deconvoluted donor and acceptor fluorescence spectra for the two cyt *c* concentrations measured illustrates how acceptor and donor emission mirror one another over the 4 s measured. The reduction of ferric cyt *c* by equimolar NADPH‐reduced CPR‐DA (Fig. 9D) is similar to that for the unlabelled sample, with second‐order rate constants of 1.01 ± 0.01 and 0.34 ± 0.02 μm ^−1^·s^−1^ for the first and second kinetic phases, respectively. These cyt *c*‐dependent rate constants illustrate that the oxidative kinetics of CPR are unaffected by the bulky fluorophores bound to it. The relative D:A emission, shown in Fig. 9C, demonstrates that the two domains slowly open after the reduction of cyt *c* by NADPH‐reduced CPR has occurred (Fig. 9E).

## Discussion

*Homo sapiens* cytochrome P450 reductase (CPR) plays an important physiological role in the transfer of electrons from NADPH to many single electron‐accepting haem‐containing proteins including CYPs, and is also commonly used as a model system to understand the mechanism of ET in the diflavin oxidoreductase enzyme family 42. CPR is thought to exist in a mixture of dynamically exchanging conformational states that may be described in terms of an energy or conformational landscape 14, 36, 37, 38. Although real‐time analysis of the conformational landscape during the complete catalytic cycle of CPR (or any enzyme system) is lacking, previous studies using fluorescently labelled CPR have suggested that domain dynamics are kinetically linked to the reductive half‐reaction of the enzyme 14. In the present study, we monitored domain dynamics during the complete catalytic cycle of CPR, while also addressing technical concerns relating to the quenching of extrinsic dye fluorescence by the flavin cofactors observed previously 14. By using a pair of fluorescent dyes (Cy5 and Alexa 750) that are active in the IR region and covalently attached to wild‐type CPR, we were able to monitor conformational change during both the reductive and oxidative half‐reactions of CPR with NADPH and cyt *c*, respectively.

There are no detectable changes in fluorescence emission from either Cy5 or Alexa 750 in CPR‐DA that suggest domain re‐orientation when oxidized CPR interacts with either ferric or ferrous cyt *c* (Fig. 2F). This suggests that cyt *c* does not bind to oxidized CPR and/or that the interactions between these two proteins upon binding do not cause significant structural rearrangement of CPR. A recent NMR study of the interaction between the FMN domain of CPR and cyt *c* has demonstrated that transient dynamic complexes are formed between the two proteins 39. Taken together, these data are consistent with a model whereby ET from CPR to cyt *c* occurs in a diffusion‐controlled manner through formation of a collisional complex that does not require domain re‐organization in CPR.

UV‐Vis double‐mixing stopped‐flow experiments showed that the oxidative half‐reaction kinetics of CPR with cyt *c* are dependent on both the oxidation state of CPR (Fig. 5) and the (ageing) time delay between addition of NADPH and cyt *c* to CPR (Fig. 4). During these experiments, an initial burst phase is followed by slower pseudo‐steady‐state turnover 23, 39. We focus here on the burst phase kinetics, but note that the apparent rate constants extracted may be convoluted with contributions from multiple turnovers. When CPR is mixed with equimolar NADPH, subsequent cyt *c* reduction occurs in two phases, which are both strictly second‐order with respect to cyt *c* concentration (Fig. 5A). These kinetics are also dependent on the ageing time (Fig. 4), suggesting that the rate of inter‐protein ET is limited by the diffusion‐controlled encounter of CPR and cyt *c*, and that the multiple kinetic phases and dependence on ageing time may arise from different oxidation and/or conformational states of CPR.

When CPR is reduced by multiple equivalents of NADPH, the cyt *c* reduction kinetics are more complex, again showing two phases that now exhibit saturation behaviour (Figs 4E and 5C), consistent with the inter‐protein ET becoming rate‐limited by a preceding chemical step. Upon reduction by two or four NADPH equivalents, the faster saturating rate constants (*k*_1for_, Table 1) are comparable to inter‐flavin ET rate constants determined previously using temperature‐jump 28 and laser flash photolysis methods 29, 30. It is likely that turnover under saturating NADPH is (partly) rate‐limited by inter‐flavin ET in CPR. Indeed, with a 20‐fold excess of NADPH, cyt *c* reduction by CPR becomes first‐order, and the burst‐phase electron flux determined using stopped‐flow experiments is similar to *k*_cat_ (Fig. 6).

Taken together, the oxidative half‐reaction kinetics determined here strongly suggest that inter‐protein ET occurs via a collisional complex despite exhibiting saturation behaviour, and the pseudo‐first‐order (saturation) kinetics are likely to arise due to rate‐limiting inter‐flavin ET in CPR. It is also noteworthy that, while the CPR to cyt *c* ET appears fastest when CPR is reduced with only one or two electrons (Fig. 5), a comparison of the apparent second‐order rate constants (*k*_for_/*K*_S_ for the saturating cases; Table 1) shows that the diffusion‐controlled rate of inter‐protein ET is unlikely to be dependent on CPR redox state. It is not obvious why two kinetic phases are observed in all experiments, but these may arise from different rates of ET from the CPR FMN semiquinone and hydroquinone species to cyt *c*, for example, as CPR is not completely four‐electron‐reduced by saturating NADPH 24.

Use of the FRET pair Cy5 and Alexa 750 improves on previous work 14 by exploiting fluorophores that absorb and emit in the near‐IR region, thus minimizing spectral overlap with, and fluorescence quenching by, the CPR flavin cofactors. However, the limitations to this approach include the relatively low quantum yield of these fluorophores (0.28 for Cy5 and 0.12 for Alexa 750), and the poor red sensitivity of fluorescence detectors. Nevertheless, the relative magnitude of donor and acceptor emission changes and the FRET efficiency (D:A fluorescence emission ratio) are comparable to those in our previous study using green/red fluorophores 14. While the domain dynamics in CPR are likely to be complex, involving multiple conformers 36 that may dynamically interconvert via ‘rotating’ and ‘swinging’ motions 43, FRET experiments only observe elements of these dynamics, as they report on the distance between two points on the protein. In CPR, the two fluorophore labelling sites have been suggested to move further apart, thus decreasing the FRET efficiency, when the protein ‘closes’ to form more compact conformation(s) 14. Here, we interpret these FRET data similarly to 14 within the context of a simplified two‐state model involving ‘open’ and ‘closed’ conformations’, but do not mean to infer that multiple conformations of the enzyme are not present.

Upon reduction of CPR‐DA with NADPH, the enzyme appears to close (Figs 6 and 7). This closing occurs with comparable kinetics to the UV‐Vis absorbance changes associated with the reduction of CPR to the di‐semiquinone state (Table 2), suggesting that flavin reduction and conformational change are kinetically coupled. This closing event was not previously observed in FRET experiments of fluorescently labelled CPR 14, and was uncovered here due to the increased time resolution and signal‐to‐noise ratio that we were able to achieve using the Cy5/Alexa 750 dye pair. The closing of CPR on this time scale probably enables efficient ET between FAD and FMN, and may be driven by NADPH binding rather than flavin reduction, as NADP^+^ binding has been shown to cause CPR to form more ‘closed’ conformations 14, 36. Upon further reduction of CPR‐DA by excess NADPH, the enzyme re‐opens with similar kinetics to conversion of the di‐semiquinone state to a further/fully reduced state (Fig. 7). The conformation(s) of this new ‘open’ state may differ from the fully oxidized state, but result in a similar mean dye–dye separation and thus similar FRET efficiency. The kinetic coupling of flavin reduction and protein ‘opening’ in CPR was observed previously 14, and suggests that redox change may act as a driver for conformational change in this enzyme. This opening event may have a functional consequence if it makes the FMN more accessible to partner cytochromes (assuming that ET from CPR to cyt *c* occurs only from the FMN), thus facilitating faster inter‐protein ET.

To investigate CPR dynamics during the oxidative half‐reaction, two‐electron‐reduced CPR‐DA was mixed with cyt *c* in a double‐mixing stopped‐flow FRET experiment. Under these conditions, CPR is observed to open with kinetics that are slower than the cyt *c* reduction monitored by UV‐Vis absorption, appearing to be first‐order with respect to cyt *c* concentration, (Fig. 9 and Table 2). It had previously been suggested that ET from a closed form of CPR would be slow, based on analysis of a disulfide locked variant of the enzyme 38. However, this variant was also impaired in its ability to transfer electrons between the flavin cofactors, suggesting that it may be locked in a non‐productive conformation by the disulfide cross‐link 38 and/or that efficient inter‐flavin ET requires conformational sampling 36. The data in Figures 4 and 5 show that cyt *c* is reduced by two‐electron‐reduced CPR, which consequently must be in a ‘closed’ conformation (Fig. 8). Unfortunately, we were unable to measure significant differences in FRET within CPR upon mixing fully reduced CPR‐DA with cyt *c*, as the FRET efficiencies of the fully reduced and re‐oxidized CPR samples are similar (Figs 7 and 8). However, it is quite likely that four‐electron‐reduced CPR also undergoes some form of structural rearrangement during/after reduction of cyt *c*. Further, Fig. 4C shows that inter‐protein ET is fastest at short ageing times when CPR is two‐electron‐reduced but still in an open conformation. While inter‐protein ET between CPR and cyt *c* has not been evolutionarily optimized (they are not physiological partners), it appears that inter‐protein ET to cyt *c* may be faster when CPR is in an open conformation. There may be differences in the mechanism and/or control of inter‐protein ET to physiological partner proteins such as CYPs, and it is possible that CPR may only reduce these proteins when in an open conformation, i.e. ‘leaky’ inter‐protein ET from closed forms of CPR is minimized under physiological conditions.

The use of single‐ and double‐mixing UV‐Vis and FRET stopped‐flow experiments provides new insight into the dynamics and chemistry of the full catalytic cycle of the reaction of CPR with cyt *c*, which is shown diagrammatically in Figs 7D and 8D for the reductive half‐reaction and Fig. 9E for the oxidative half‐reaction. In this dynamic model of catalysis, NADPH binding and/or FAD reduction cause CPR to adopt a more compact ‘closed’ conformation. The subsequent slower inter‐flavin ET may be accompanied by further structural rearrangement, with some two‐electron‐reduced species adopting more compact conformations. Further reduction of CPR by a second NADPH is accompanied by conformational changes resulting in a more open form of the enzyme, consistent with our previous study 14. Inter‐protein ET to cyt *c* may probably occur from both open and closed conformations of one‐, two‐, three‐ and four‐electron‐reduced CPR, but ET may be fastest from more open conformations, and conformational rearrangement of CPR subsequent to the inter‐protein ET event may be relatively slow.

### Concluding remarks

**Figure 10:**
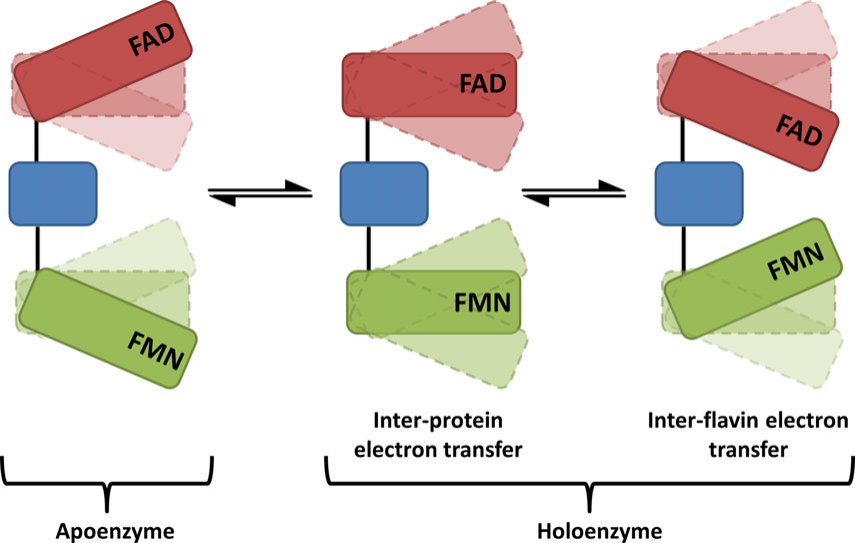
Schematic representation of the conformational equilibria of CPR. The NADP/FAD‐binding domain is shown in red, the FMN‐binding domain is shown in green, and the connecting domain is shown in blue. The NADP ^+^‐free form of CPR adopts predominantly more open conformations, with relatively larger distances between the flavin cofactors. Upon coenzyme addition (NADP ^+^/NADPH), oxidized CPR makes a transition to more compact forms, with relatively shorter inter‐flavin distances. Transfer of a hydride anion from NADPH to FAD causes a shift in the conformational sub‐states to predominantly more compact CPR conformations. These compact forms favour electron transfer from FADH_2_ to FMN (i.e. short electron transfer distances relative to more open conformations). When CPR is reduced with excess NADPH, an additional opening phase is observed after initial closing triggered by (stoichiometric) NADPH reduction. This opening allows the enzyme to transfer electrons rapidly from the FMN domain of CPR to partner proteins. Electron transfer between cofactors and partner proteins may occur in multiple conformational states. However, the model implies that these reactions will be more rapid in selected conformational sub‐states in which donor–acceptor distances are shortened.

The model presented in this work (Fig. 10) provides a structural framework to describe the conformational control of inter‐ and intra‐protein ET in CPR that is likely to be applicable to all mammalian diflavin oxidoreductases, given the structural and mechanistic similarities within this enzyme family. The study emphasizes the importance of protein dynamics in intra‐ and inter‐protein electron transfer, and establishes a methodology for real‐time analysis of structural changes throughout the catalytic cycle of complex redox proteins.

## Experimental procedures

### Materials

All reagents were analytical grade and were purchased from Sigma‐Aldrich (Gillingham, Dorset, UK), unless otherwise stated.

### Recombinant protein expression and purification

*Homo sapiens* cytochrome P450 reductase (CPR), lacking the 60 amino acid membrane‐binding N‐terminus, was expressed from plasmid pET15b (Millipore, Watford, Hertfordshire, UK) in *Escherichia coli* BL21 (DE3) pLys (New England BioLabs, Hitchin, Hertfordshire, UK), and purified by DEAE–Sepharose anion exchange chromatography followed by nickel–Sepharose affinity chromatography, as described previously 40, 41. Prior to use, CPR was oxidized using a few grains of potassium ferricyanide, and immediately passed through an Econo‐Pac 10DG desalting column (Bio‐Rad, Hemel Hempstead, UK) to remove surplus oxidant. The oxidized CPR concentration was determined by absorption spectroscopy using an absorption coefficient (ε) of 22 mm ^−1^·cm^−1^ at 454 nm 22.

### Extrinsic fluorophore labelling

Maleimide labelling sites on CPR have previously been identified by mass spectrometry as C228, C472 and C566 14. To label CPR, fluorescent dyes were incubated with the enzyme in 50 mm potassium phosphate buffer (pH 7.0) at room temperature for 5 h. The dyes that were used to label CPR were Cy5 mono‐maleimide (Cy5, GE Healthcare, Little Chalfont, UK) and Alexa Fluor C_5_ 750 maleimide (Alexa 750, Life Technologies, Paisley, UK). For labelling with a 1 : 1 ratio of Cy5 to Alexa 750, 10 μm of CPR was incubated with 200 μm of both fluorophores. Following labelling, the unreacted dyes were removed from the labelled protein by passing samples through a desalting column (Econo‐Pac 10DG desalting column; Bio‐Rad, Hemel Hempstead, UK).

### Static fluorescence studies

Fluorescence emission spectra were recorded on an Edinburgh Instruments (Livingston, West Lothian, UK) FLS920 fluorometer equipped with double excitation and emission monochromators, a red‐sensitive cooled photo‐multiplier detector, and a 450 W xenon arc lamp. Spectra were recorded using 0.5 nm excitation and 5 nm emission slit widths in 1 mL fluorescent quartz cells (Starna Scientific Ltd, Hainault, UK) with a 10 mm excitation path length. Fluorescence emission data were collected at 25 °C in 50 mm potassium phosphate buffer (pH 7.0) with approximately 1 μm of CPR.

### Stopped‐flow studies and data fitting

Kinetic studies were performed using a SX20 stopped‐flow spectrometer (Applied Photophysics Ltd, Leatherhead, Surrey, UK) housed within an anaerobic glove box (Belle Technology, Weymouth, Dorset, UK, < 2 p.p.m. O_2_). Experiments were performed in 50 mm potassium phosphate buffer (pH 7.0) at 25 °C. Buffers were de‐gassed by bubbling with oxygen‐free nitrogen before placing in the glove box, where they were left overnight to remove all traces of oxygen. The reductive half‐reaction of CPR was monitored at 454 or 600 nm, as previously described 22. Reactions were initiated by mixing either an equimolar amount of NADPH or a 20‐fold stoichiometric equivalent of NADPH with 15 μm CPR (final concentration), unless stated otherwise. To follow the oxidative half‐reaction of CPR, the haem β‐band of bovine heart cyt *c* was monitored at 550 nm (Δε_550_ = 21.1 mm ^−1^·cm^−1^). A double‐mixing stopped‐flow method was used to study the oxidative half‐reaction kinetics of CPR.

For both the reductive and oxidative half‐reactions, monitored by UV‐Vis stopped‐flow spectroscopy, 4–12 transients were recorded for each experiment. Individual transients were fitted to Eqn (1), where *A*_*i*_ is the amplitude and *k*_*i*_ is the rate constant of the *i*th exponential component, and Δ*A* is the total amplitude change:

(2) $$\Delta A=\Sigma_{i\;=\;1}^{n}A_{i}\exp\left(-k_{i}t\right)$$

The results of these experiments are presented as mean rate constants ±1 standard deviation.

Fluorescence emission stopped‐flow transients monitoring both the the donor and acceptor were recorded by dual‐channel detection, using a R1104 red‐sensitive photomultiplier detector (Applied Photophysics, Leatherhead, Surrey, UK) for the acceptor channel. To monitor donor and acceptor fluorophores, 670 nm bandpass and 750 nm highpass filters (Thor Labs, Ely, UK), respectively, were used. All experiments were performed using approximately 1 μm CPR (final concentration) to ensure no inner filter effects were present. Between 9 and 15 or 20 and 40 repeats were performed for the donor and acceptor fluorophores, respectively. Due to the low quantum yield of the dyes, the transients were averaged and smoothed using a five‐point moving average. These fluorescent transients were fitted to Eqn (1). To extract the fluorescence change associated with FRET alone, the percentage of emission from CPR‐D was subtracted from the percentage emission of the corresponding fluorophore in a FRET pair (CPR‐DA); this was performed due to quenching of the donor emission by aromatic amino acids, cofactors and the flavin semiquinone absorbance feature at 600 nm. Rate constants for fluorescence data are presented with a standard error of fit. Stopped‐flow control studies indicated that NADPH (1 mm) did not reduce the FRET dyes (1 μm) directly over the time scale of the experiments (10 s) in absence of CPR/cyt *c* (data not shown).

Rate constants for the UV‐Vis stopped‐flow kinetics were plotted against cyt *c* concentration, and fitted to either a linear function (one‐step model) or a hyperbolic function (two‐step model) (Eqn (3)):

(3) $$k_{\mathrm{obs}}=k_{\mathrm{rev}}+\frac{k_{\mathrm{for}}\left[\mathrm{cyt}\;c\right]}{K_{S}+\left[\mathrm{cyt}\;c\right]}$$

Equation (2) allowed the kinetic parameters [rate of reverse reaction (*k*_rev_), rate of forward reaction (*k*_for_) and substrate saturation constant (*K*_*S*_)] to be determined for the two‐step model of cyt *c* reduction by reduced CPR.

Steady‐state turnover of CPR was followed by observing the reduction of cyt *c* at 550 nm under saturating conditions of NADPH (200 μm). At least five traces were recorded for each cyt *c* concentration. The *K*_*m*_ and *k*_cat_ values for cyt *c* were determined by fitting data to the Michaelis–Menten equation.

## Author contributions

N.S.S. and S.H. conceived and designed the project; T.M.H. acquired the data; all authors analysed and interpreted data, and wrote the paper.
